# Neohesperidin Dihydrochalcone Alleviates Lipopolysaccharide‐Induced Vascular Endothelium Dysfunction by Regulating Antioxidant Capacity

**DOI:** 10.1002/iid3.70107

**Published:** 2024-12-19

**Authors:** Yuxin Nong, Junquan Lu, Danqing Yu, Xuebiao Wei

**Affiliations:** ^1^ Department of Cardiology, Guangdong Provincial Key Laboratory of Coronary Heart Disease Prevention, Guangdong Cardiovascular Institute, Guangdong Provincial People's Hospital Guangdong Academy of Medical Sciences Guangzhou China; ^2^ Shantou University Medical College Shantou China; ^3^ Department of Geriatric Intensive Medicine, Guangdong Provincial Geriatrics Institute Guangdong Provincial People's Hospital, Guangdong Academy of Medical Sciences Guangzhou China

**Keywords:** endothelial dysfunction, inflammation, neohesperidin dihydrochalcone, oxidative stress, sepsis

## Abstract

**Background:**

Endothelial dysfunction is one of the important mechanisms of organ and tissue damage in sepsis. In this study, we evaluated the effects of neohesperidin dihydrochalone (NHDC) on lipopolysaccharide (LPS)‐induced vascular dysfunction and explored the potential mechanisms.

**Methods:**

In vivo, we assessed vascular leakage in mice by injecting Evans blue dye. In vitro, cell counting kit‐8 (CCK‐8) assay and flow cytometry were used to assess the activity of HUVEC and apoptosis. The effect of LPS on HUVEC barrier was assessed using FITC‐extend membrane assay. The adhesion ability of HUVEC was tested by THP‐1 cell adhesion assay. The antioxidant capacity of cells was measured by detecting the level of mitochondrial membrane potential, ROS, and content of CAT, SOD, GSH, and MDA within the cells. Furthermore, the release of endothelial IL‐1β, IL‐6, and TNF‐α were detected by ELISA, and the expression level of TAK1, ERK1/2, and NFκB were detected by western blot.

**Results:**

Treatment with NHDC effectively alleviated LPS‐induced endothelial permeability and organ damage by reducing reactive oxygen species production and enhancing the antioxidant response. Further investigation suggested that NHDC may exert its protective effects by inhibiting the release of IL‐1β, IL‐6, and TNF‐α, and by decreasing the phosphorylation of key inflammatory signaling molecules, including transforming growth factor‐β‐activated kinase 1 (TAK1), extracellular signal‐regulated kinases 1/2 (ERK1/2), and nuclear factor kappa B (NFκB).

**Conclusions:**

Our study indicate that pretreatment with NHDC may provide protection against LPS‐induced vascular dysfunction by reducing oxidative stress and activation of inflammatory signaling pathways.

## Graphical abstract

NHDC may play a protective role in LPS‐induced vascular dysfunction by reducing oxidative stress and activation of inflammatory signaling pathways.

## 1 Introduction

Endothelial dysfunction plays a pivotal role in the development of organ dysfunction during sepsis. In sepsis, pathogens disrupt the structural and functional integrity of the endothelium, compromising its protective barrier function and leading to leakage of fluid from blood vessels into extravascular spaces [1, 2]. The endotoxin produced by Gram‐negative bacteria is the main contributor to vascular endothelial injury. It activates the inflammatory response of endothelial cells via toll‐like receptors, disrupting immune signaling and triggering the release of inflammatory mediators. Concurrently, it compromises the barrier function of endothelial cells, allowing fluid to leak from blood vessels into the extravascular spaces. The intravascular hypovolemia that ensues further exacerbates reduced perfusion and hypoxia in organs and tissues [3].

Neohesperidin dihydrochalcone (NHDC) is a widely used, safe, low‐calorie, nonnutritive artificial flavoring agent derived from flavanone neohesperidin [4, 5]. Previous studies have shown that NHDC can reduce the level of LPS‐induced inflammatory factors in vivo, regulate the oxidative phosphorylation of mitochondria, and improve glucose and lipid metabolism in rats induced by a high‐fat diet [6, 7]. Further studies revealed that NHDC can inhibit subcutaneous fat accumulation via the PI3K/AKT/mTOR pathway to regulate fatty acid uptake [8]. Recent report show that NHDC alleviates sepsis‐induced acute kidney injury by inhibiting apoptosis of renal tubular epithelial cells and inflammatory pathways [9]. Furthermore, it is reported that NHDC inhibits the activation of myeloid differentiation factor 88 signaling to reduce LPS‐induced liver injury and macrophage inflammation [10].

In this study, we used LPS to simulate the endotoxin environment of sepsis and examined the effect of pretreatment on LPS‐induced vascular dysfunction using both in vivo and in vitro experiments.

## 2 Methods

### 2.1 Chemicals and Reagents

NHDC (cat # HY‐N0154), fluorescein isothiocyanate‐labeled dextran (FITC dextran MV10000, cat # HY‐128868), Evans Blue (cat # HY‐B1102), and BAY 11‐7082 (cat # HY‐13453) were purchased from Med Chem Express (New Jersey, United States). Lipopolysaccharides (LPS, *Escherichia coli* 055: B5) were purchased from Sigma‐Aldrich (Missouri, USA), and Endothecal Cell Medium (ECM, cat # 1001) was purchased from ScienCell (San Diego, California, USA). RPMI 1640 culture medium (cat # 11875101) was purchased from Thermo Fisher Scientific Inc. (Massachusetts, USA). Fetal bovine serum (FBS, cat# 164210‐50) was purchased from Procell Life Science & Technology Co. Ltd. (Wuhan, China). Carboxymethyl cellulose sodium (CMC Na, cat# C804628) and formamide (cat# F809511) were purchased from Macklin (Shanghai, China). Bovine serum albumin (BSA, cat# BP0005), paraformaldehyde (cat# DF0135), and MagZol Reagent (Trizol Reagent, cat# R4801‐01) were obtained from Angen Biotech Co. Ltd. (Beijing, China). Calcein AM (cat# C2012), mitochondrial membrane potential assay kit with JC‐1 (cat# C2006), BCA protein assay kit (cat # P0010), SDS‐PAGE Sample Loading Buffer (cat # P0015L), reactive oxygen species (ROS) Assay Kit (cat # S0033S), and quickBlock Blocking Buffer for Immunol Staining (cat # P0260) were purchased from Beyotime Biotechnology (Shanghai, China). Superoxide Dismutase (SOD) assay kit (cat # A001‐3‐2), reduced glutathione (GSH) assay kit (cat # A006‐2‐1), and Malondialdehyde (MDA) assay kit (cat # A003‐1‐2) were purchased from Nanjing Jiancheng Bioengineering Research Institute (Nanjing, China). Dylight 488, Goat Anti‐Rabbit IgG (H + L) (Cat # E032220) was purchased from EarthOx Life Sciences (California, USA). Rabbit anti‐transforming growth factor‐β‐activated kinase 1 (TAK1) (cat # AF7616), rabbit anti‐β‐actin (cat # AF7018), and horseradish peroxidase (HRP)‐coupled second antibody (cat # S0001) were purchased from Affinity Biosciences (Jiangsu, China). Rabbit anti‐nuclear factor kappa B (NF‐κB)‐p65 (cat # ab76302) and intercellular adhesion molecule‐1 (ICAM‐1) (cat# ab109361) were purchased from Abcam (Cambridge, UK). Rabbit anti‐phospho‐TAK1 (cat# 9339S), rabbit anti‐phospho‐extracellular signal‐regulated protein kinases 1 and 2 (ERK1/2; cat # 4370S), and rabbit anti‐ERK1/2 (cat# 137F5) were purchased from Cell Signaling Technology (Massachusetts, USA). All chemical reagents, unless otherwise specified, were analytically pure.

### 2.2 Experimental Animal

One hundred C57BL/6 male mice (8‐week‐old, weighing 20–25 g) were purchased from the Guangdong Medical Laboratory Animal Center. Prior to the experiments, all mice were acclimatized for 3 days in a specific pathogen‐free (SPF) environment (temperature: 20°C–24°C, relative humidity: 50%–60%). During this period, they had ad libitum access to food and water, and were maintained on a 12‐hour light/dark cycle. These mice were randomly divided into four groups, with 25 mice in each group: (1) control group, (2) NHDC group, (3) LPS group, and (4) LPS + NHDC group. With reference to previous studies [9, 10], mice in the NHDC and LPS + NHDC groups were given an oral gavage of NHDC at 100 mg/kg for 5 days. NHDC was dissolved in 0.5% carboxymethyl cellulose sodium (CMC‐Na), a commonly used cosolvent to facilitate its dissolution. Mice in the control and LPS groups received an equivalent volume of 0.5% CMC‐Na. Following the oral treatment,mice in LPS and LPS + NHDC groups were injected intraperitoneally with a dose of 15 mg/kg of LPS that dissolved in physiological saline [11, 12, 13, 14]. After experiments, all mice were euthanized, and organ samples were collected for further analysis. The study protocol was reviewed and approved by the Ethics Committee of Guangdong Provincial People's Hospital (approval number: KY2023‐133‐02).

### 2.3 Cell Culture and Treatment

Human umbilical vein endothelial cells (HUVEC) and human monocyte‐derived THP‐1 cells were purchased from the American Type Culture Collection (ATCC). HUVECs were cultured in endothelial cell medium (ECM) supplemented with 10% fetal bovine serum (FBS). Cells were seeded in 6‐well plates, 12‐well plates, or 96‐well plates (NEST, China). In the NHDC and LPS + NHDC groups, HUVECs were pretreated with NHDC (25 μM) for 6 hours, followed by treatment with lipopolysaccharide (LPS) at 10 mg/mL for 24 hours. Cells in the LPS and control groups were not treated with NHDC. THP‐1 cells were cultured in RPMI 1640 medium containing 10% fetal bovine serum (Gibco, USA) and supplemented with 1% penicillin‐streptomycin. All cells were maintained in a 37°C incubator with 5% CO_2_. After the experiments, all cells and supernatants were collected for subsequent experiments.

### 2.4 Broncho‐Alveolar Lavage Fluid (BALF)

We assessed the degree of inflammation and edema in the lungs by BALF [15]. Briefly, at the end of the experimental treatment, mice were euthanized, and the chest cavity was exposed. One milliliter of phosphate‐buffered saline (PBS) was then injected into the lungs via the bronchus using a 1 mL syringe. After the injection, the lavage was slowly withdrawn, and the procedure was repeated three times. BALF collection was considered successful if the ratio of withdrawn fluid to injected fluid was greater than 60%. Finally, the total protein concentration of BALF was measured using the BCA assay.

### 2.5 Histological Analysis

Mouse organs were first rinsed with normal saline to remove blood from the surface and then blotted dry with filter paper. The tissues were subsequently fixed in 4% paraformaldehyde solution. Then, the organs were embedded with paraffin wax and cut into 4‐μm‐thick slices using a pathological Microtome (Shandon Finesse 325, Thermo Scientific). The sections were dewaxed in a dewaxing agent for 10 minutes and rehydrated through a graded ethanol series (100%, 95%, 85%, and 75%), with each step lasting 5 minutes. Finally, hematoxylin and eosin (H&E) staining were performed. Under a light microscope (Nikon, Japan), observations focused on cellular morphology, tissue contour integrity, the degree of inflammatory cell infiltration, and the composition of intercellular substances.

### 2.6 Vascular Permeability Analysis In Vivo

Evans blue dye was used to evaluate vascular permeability in vivo. Evans blue, an azo dye with a high affinity for albumin, forms a stable complex with albumin in the bloodstream. Under normal conditions, albumin does not readily cross the vascular barrier; however, when vascular permeability increases, the Evans blue–albumin complex leaks into surrounding tissues [16]. Six hours after intraperitoneal administration of LPS, mice were injected via the tail vein with 200 μL of 0.5% Evans blue solution. Fifteen minutes later, the mice were euthanized, and their organs were collected. The surface water was carefully removed with filter paper, and each tissue sample was incubated with 500 μL of formamide in a thermostatic oscillator at 55°C for 48 hours to extract the Evans blue dye. Then, the tissue fragments were precipitated by centrifugation (900 g × 10 min). The absorbance of the supernatant, containing formamide and Evans blue, was measured at 610 nm using a universal microplate reader (TECAN Infinite 200 PRO, Switzerland). The amount of Evans blue extravasation was quantified as nanograms per milligram of tissue using a standard curve.

### 2.7 Cell Viability

The viability of HUVECs under different NHDC concentrations was assessed by cell counting kit‐8 (CCK‐8) (Dojindo, Japan). Initially, HUVEC cells were seeded in 96‐well plates and treated with varying concentrations of NHDC for 24 h. Subsequently, CCK‐8 solution was added to each well following the manufacturer's protocol. Absorbance at 450 nm was measured using a universal microplate reader (Infinite 200 PRO TECAN, Switzerland).

### 2.8 Cell Permeability Test In Vitro

HUVEC cells were seeded evenly into the upper chambers of a transwell with small holes (0.4 μm in diameter) within a 6‐well plate (Corning, New York, USA). Confluent monolayers were used to evaluate barrier function. In short, following treatment, 1 μg/mL fluorescein isothiocyanate‐labeled dextran (FITC‐dextran) was added to the upper chamber, and 600 μL of medium was added to the lower chamber. After a 1‐hour incubation in the dark, 100 μL of medium from both the upper and lower chambers was transferred to 96‐well plates. Fluorescence intensity was measured using a universal microplate reader with excitation at 488 nm and emission at 525 nm (Spark Multimode Microplate Reader TECAN, Switzerland). Endothelial cell permeability in vitro was quantified as the fluorescence intensity ratio of the lower chamber to the upper chamber.

### 2.9 Endothelium‐Monocyte Cell Adhesion Experiment

THP‐1 cells were incubated with 5 μM calcein AM for 30 min to enable fluorescent labeling. Subsequently, the THP‐1 cells were washed three times with phosphate‐buffered saline (PBS), resuspended, and collected by centrifugation at 250 g for 5 minutes. These labeled THP‐1 cells were then incubated with HUVEC for 2 h. Finally, the cells were observed and counted using a fluorescent inverted microscope (Nikon, Japan).

### 2.10 Flow Cytometry

After treatment, both the supernatant and cells were collected into a 1.5 mL EP tube, centrifuged, and washed twice with precooled PBS. Annexin V‐AbFluor 488/PI fluorescent probe (Abbkine, cat # KTA0002) was then added and the mixture was incubated for 15 minutes. Finally, cell apoptosis was analyzed using FITC and phycoerythrin channels on a flow cytometer (Beckman Coulter CytoFLEX, USA).

### 2.11 ROS Detection

After treatment, the cells were washed twice with PBS. A 2',7'‐dichlorofluorescin diacetate (DCFH‐DA) probe, diluted at a ratio of 1:1000, was added, and the cells were incubated for 30 minutes at 37°C. Subsequently, the cells were washed three times with serum‐free culture medium to remove any residual extracellular DCFH‐DA. Finally, intracellular ROS levels were visualized a fluorescence microscope.

### 2.12 Biochemical Analysis

We determined the levels of total superoxide catalase (CAT), dismutase (T‐SOD), reduced glutathione (GSH), and malondialdehyde (MDA) to assess the antioxidant capacity of the cells. Following thorough washing with PBS, the cells were homogenized completely, and corresponding reagents were added for reactions. Finally, the absorbance at the appropriate wavelength was read and the concentration of the corresponding substance was calculated by using a universal microplate reader.

### 2.13 Enzyme‐Linked Immunosorbent Assay (ELISA)

The levels of interleukin (IL)‐1β, IL‐6, and tumor necrosis‐α (TNF‐α) in the supernatant were detected using an ELISA kit (Jiangsu Jingmei Biotechnology, China). According to the manufacturer's instructions, samples and labeled antibodies were incubated at 37°C for 1.5 hours in a water bath. After the incubation, the plates were washed thoroughly with the provided washing solution to remove unbound components. Color substrate was added, and the reaction was stopped after appropriate color development. Finally, the absorbance at 450 nm was measured using an enzyme marker, and cytokine concentrations were calculated based on standard curves.

### 2.14 Mitochondrial Membrane Potential (MMP) Detection

The mitochondrial membrane potential (MMP) was assessed using a JC‐1 assay kit, a fluorescent probe commonly used for detecting ∆Ψm in mitochondria [17]. When the MMP is high, JC‐1 accumulates in the matrix of mitochondria, forming a polymer (JC‐1 aggregates), which can produce red fluorescence. Conversely, When the MMP is low, JC‐1 remains in its monomeric form, emitting green fluorescence. The MMP was quantified by calculating the ratio of red (JC‐1 aggregates) to green (JC‐1 monomers) fluorescence intensity. After experimental treatment, cells were incubated with JC‐1 staining working solution at a dilution ratio of 1:200 for 15 minutes at 37°C. After incubation, the staining solution was removed, and the cells were washed thoroughly with PBS. Fluorescence was then observed and analyzed using a fluorescent inverted microscope.

### 2.15 Immunofluorescence

The cells were cultured in 24‐well plates. After exposure to the treatment, they were fixed with 4% paraformaldehyde for 15 min, washed with PBS, and incubated with QuickBlock Blocking Buffer for Immunol Staining at room temperature for 15 min. Following this, the cells were incubated overnight with an ICAM‐1 antibody at a 1:200 dilution at 4°C. Subsequently, the cells were thoroughly washed with PBS and incubated with fluorochrome‐conjugated goat anti‐rabbit IgG (H+L) at room temperature for 30 min. After a final PBS wash, the cells were observed and photographed under a fluorescence microscope.

### 2.16 Western Blot Analysis

After treatment, cells were washed three times with PBS. Subsequently, they were collected, fully lysed on ice, and centrifuged at low temperatures to extract the total cellular protein by using kit (BBproExtra, cat #BB‐31013). The protein concentration was determined using BCA protein assay kit. Proteins were denatured by boiling with SDS‐PAGE Sample Loading Buffer at 100°C for 5 minutes. Electrophoresis at 4°C with primary antibodies separation was performed on 10%–15% SDS‐PAGE gel, and the proteins were subsequently transferred to a polyvinylidene fluoride membrane. The membrane was sealed with 5% BSA at room temperature for 1 h and incubated overnight at 4°C with primary antibodies. The next day, the membrane was thoroughly washed with TBST solution (Tris‐buffered saline with Tween 20) and incubated with horseradish peroxidase (HRP)‐conjugated second antibody at room temperature for 1 hour, according to the instructions of the manufacturer. Finally, protein bands were visualized using a gel imaging system (ImageQuant LAS500 Cytiva, USA).

### 2.17 Statistical Analysis

Continuous data are expressed as mean ± standard deviation (SD) and compared by using the Student *t*‐test or Mann–Whitney test. Survival of mice was expressed as Kaplan–Meier curves and compared by the log‐rank test. A two‐sided *p*‐value of 0.05 was considered statistically significant. All experiments were replicated at least three times. Statistical analyses were analyzed using GraphPad Prism software 9.0 (California, USA).

## 3 Results

### 3.1 NHDC Alleviates LPS‐Induced Organ Damage and Improved Survival in Mice

**Figure 1:**
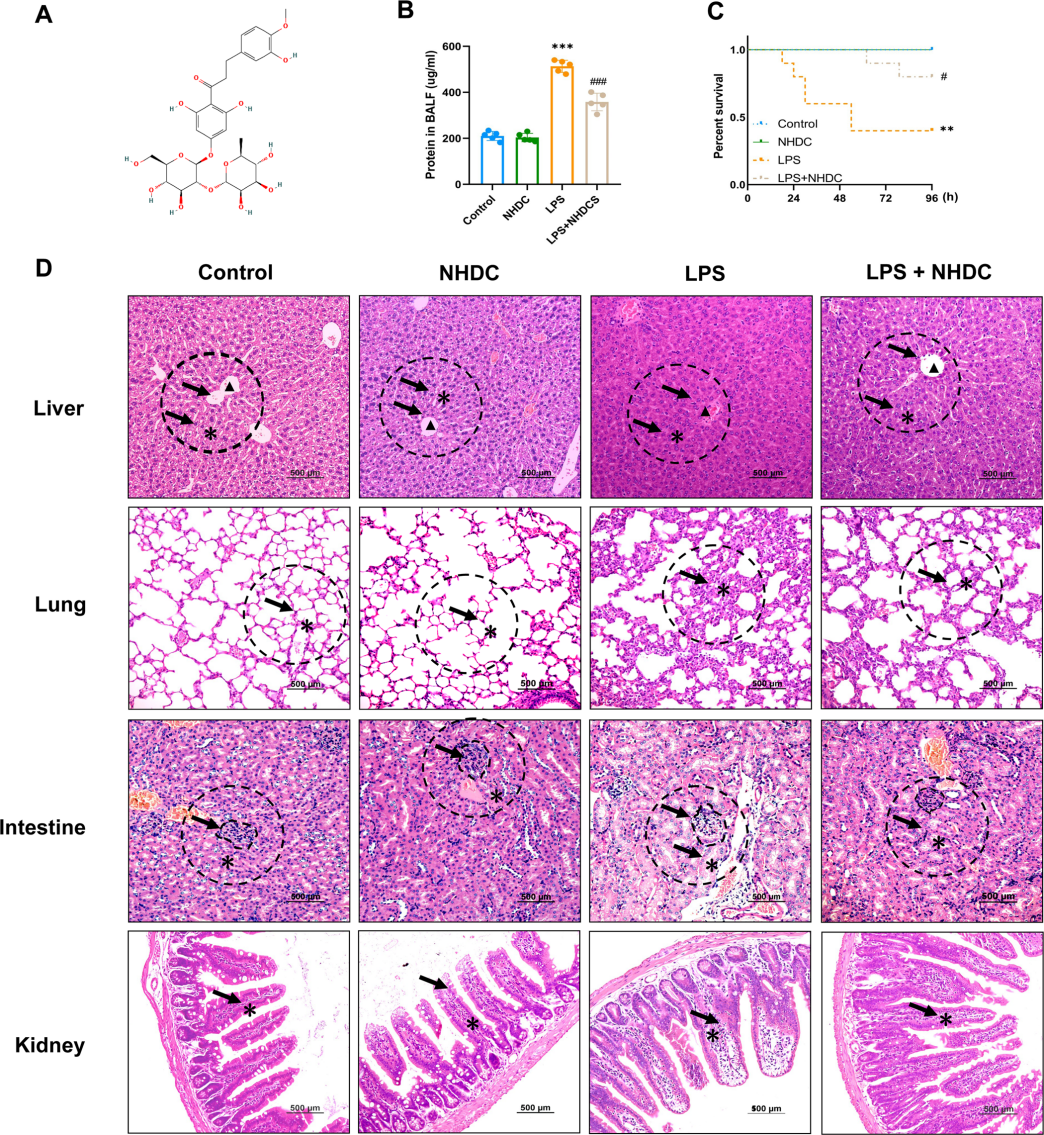
Effect of NHDC on LPS‐induced organ damage and survival in mice. (A) Schematici llustration of the chemical molecular structure of NHDC. (B) Protein concentration in the broncho‐alveolar lavage fluid (*n* = 5). (C) Kaplan–Meier survival curves for mice after LPS intraperitoneal injection (*n* = 10). (D) Representative histopathological sections stained with hematoxylin and eosin. The black arrows indicate the tissues with pathological changes after treatment in different groups (*n* = 4). The arrow indicates the dotted circle, which marks the classic representative area of the tissue. In the liver, * denotes the hepatic cords and sinusoids, while ▲ marks the central vein. In the lungs, * represents the alveolar structures and walls. In the kidney, the dotted circle highlights the glomerulus, and * indicates the tubular epithelial cells. In the intestine, * refers to the villi structure. Scale bar = 500 μm; *p < 0.05, **p < 0.01, ***p < 0.001, ^#^ *p* < 0.05, ^###^ *p* < 0.001; *Compared with control group, #compared with LPS group.

The chemical molecular structure of NHDC is shown in Figure 1A. We simulated the endotoxin environment of sepsis by intraperitoneally injecting LPS at a dose of 15 mg/kg into mice. Our study showed that the pretreatment with NHDC reduced protein extravasation in response to LPS intervention in BALF experiments (358.0 ± 38.0 μg/mL vs. 513.2 ± 26.2 μg/mL, *p* < 0.001) (Figure 1B). The pretreatment of NHDC can improve the survival of the mice (Figure 1C). Histopathological analysis further demonstrated severe organ damage in the LPS‐treated group. In the liver, pronounced swelling and severe congestion of hepatic sinusoids were observed, accompanied by disorganization of the liver lobule structure due to cellular edema. Pulmonary examination revealed significant inflammatory cell infiltration in alveolar tissues and thickened alveolar septa. Renal examination showed hydropic degeneration of the tubular epithelial cells, while the glomerular structure appeared swollen and poorly defined. In the intestine, the integrity of the villi was compromised, with the brush border disappearing and extensive infiltration of inflammatory cells. However, pretreatment with NHDC significantly attenuated these pathological alterations (Figure 1D).

### 3.2 NHDC Alleviates LPS‐Induced Endothelial Dysfunction in Mice

**Figure 2:**
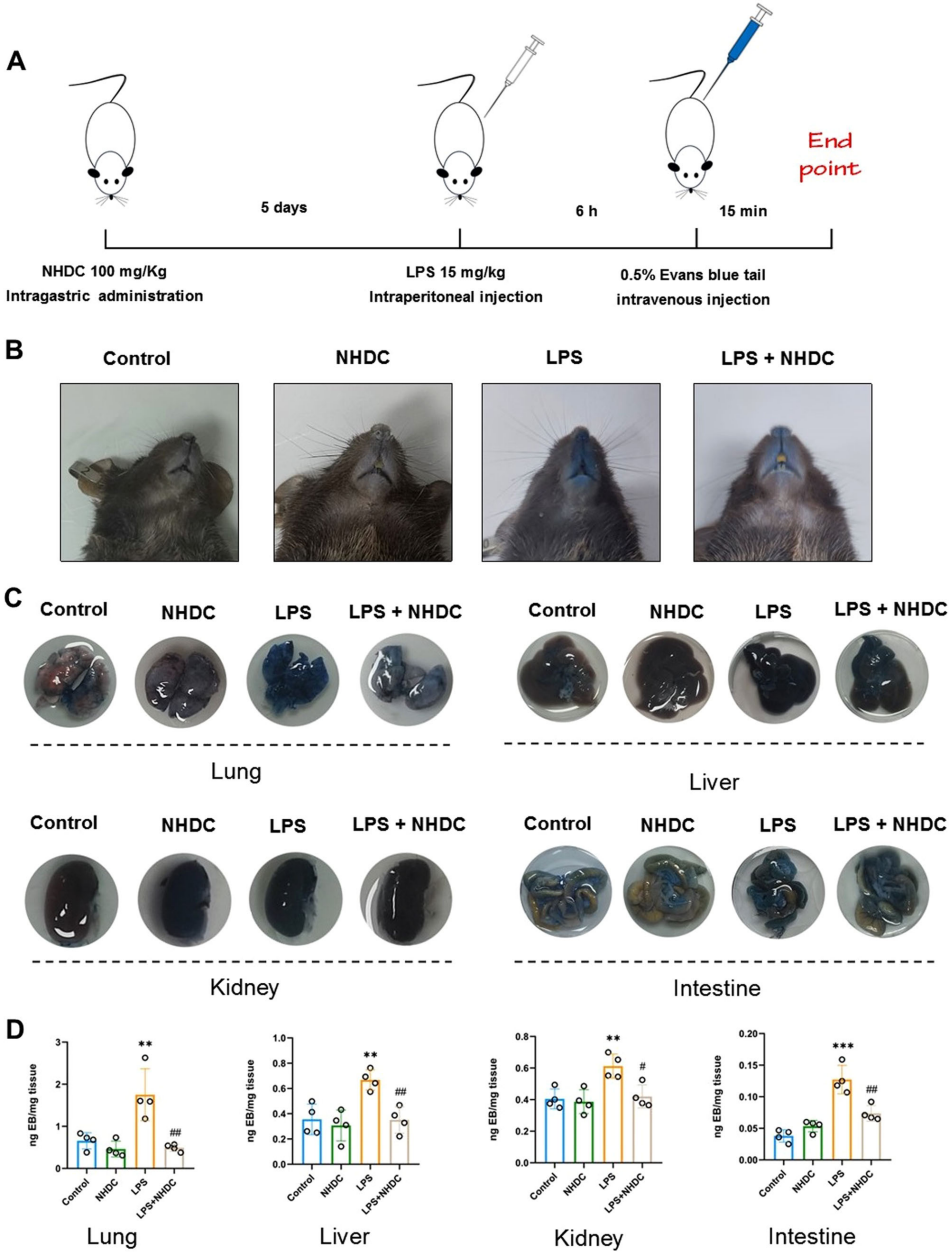
NHDC alleviates LPS‐induced vascular leakage in mice. (A) Schematic diagram of the experimental procedure. (B) Representative pictures of skin color of different groups after injection of Evans blue. (C) Representative pictures of organs of mice after injection of Evans blue solution. (D) Leakage and statistics of analysis of different mouse organs after injection of Evans blue solution (*n* = 4). **p < 0.01, ***p < 0.001, ^#^ *p* < 0.05, ^##^ *p* < 0.01; *Compared with control group, #compared with LPS group.

We further investigated the effect of NHDC on endothelial leakage induced by LPS. Evans Blue solution was injected through tail vein after intraperitoneal injection of LPS for 6 h, and then euthanasia was performed 15 min later (Figure 2A). Mice in the LPS group exhibited severe skin mucous membrane leakage compared to the control and LPS + NHDC groups, and NHDC alleviates LPS‐induced vascular leakage (Figure 2B). The contents of Evans blue between LPS + NHDC group and LPS group in lungs (0.49 ± 0.09 ng/mg vs. 1.75 ± 0.62 ng/mg, *p* = 0.007), liver (0.35 ± 0.10 ng/mg vs. 0.67 ± 0.08 ng/mg, *p* = 0.003), kidneys (0.42 ± 0.07 ng/mg vs. 0.61 ± 0.08 ng/mg, *p* = 0.011), and intestines (0.07 ± 0.01 ng/mg vs. 0.13 ± 0.02 ng/mg, *p* = 0.006) indicated that NHDC reduced blood vessel leakage. These results suggest that pretreatment of NHDC may alleviate LPS‐induced organ injury by reducing the leakage of blood vessels (Figure 2C,D).

### 3.3 In Vitro Effects of NHDC on Endothelial Cell Viability

**Figure 3:**
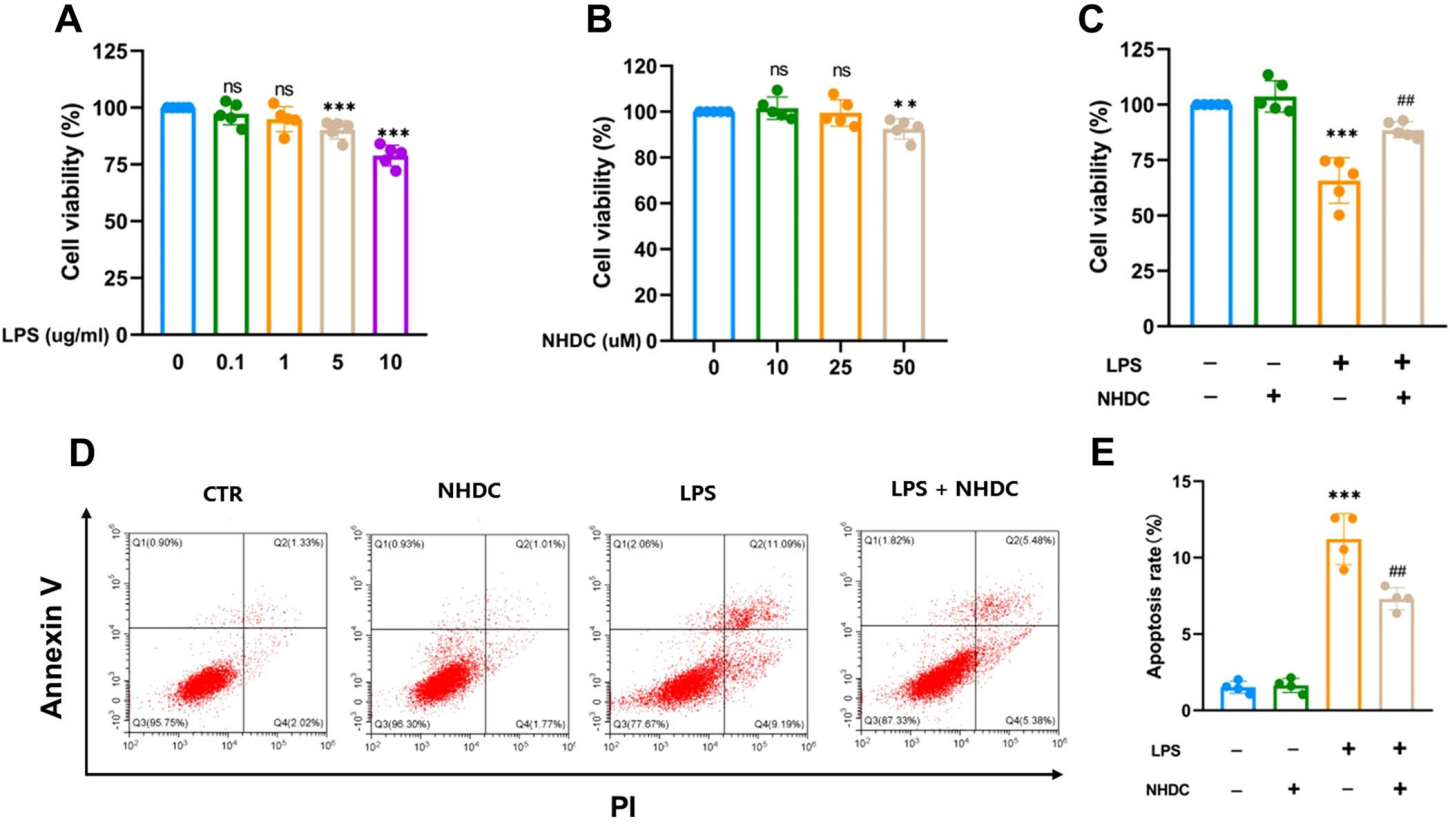
Effect of NHDC on HUVEC cell viability. (A) Effects of different LPS concentrations on HUVEC activity (*n* = 5). (B) Effects of different NHDC concentrations on HUVEC activity (*n* = 5). (C) Effect of NHDC on LPS‐induced HUVEC cell viability. (D, E) Flow cytometry results and statistical analysis of apoptotic staining with Annexin V‐FITC/PI in different groups (*n* = 4). **p < 0.01, ***p < 0.001, ^#^ *p* < 0.05, ^##^ *p* < 0.01; *Compared with control group, #compared with LPS group.

We initially assessed HUVEC damage induced by different concentrations of LPS in vitro using the CCK‐8 assay. The results showed that at a concentration of 5 µM, LPS caused mild damage to HUVECs (90.22% ± 4.05% vs. 100%, *p* < 0.001). At a concentration of 10 µM, significant damage was observed (78.91% ± 4.59% vs. 100%, *p* < 0.001), as shown in Figure 3A. Therefore, we selected 10 µM LPS to establish the subsequent in vitro vascular endothelial cell damage model. Then, we evaluated the effect of NHDC on endothelial cell viability through the CCK8 assay, and the results showed that NHDC significantly reduced HUVEC cell viability at 50 μM (92.50% ± 4.44% vs. 100%, *p* < 0.001) (Figure 3B). To ensure cell safety, we selected 25 μM as the treatment concentration for subsequent experiments. Furthermore, we found that NHDC could effectively alleviate LPS‐induced HUVEC viability injury by CCK8 assay (88.63 ± 3.58% vs. 65.74% ± 10.31%, *p* = 0.002) (Figure 3C). Apoptosis of vascular endothelial cells is an important characteristic of sepsis [18]. Furthermore, we evaluated the effect of NHDC on LPS‐induced HUVEC apoptosis by flow cytometry, and the results revealed that NHDC mitigates the LPS‐induced apoptosis in HUVEC (Figure 3D,E).

### 3.4 In Vitro Effects of NHDC on Endothelial Cell Permeability and Adhesion of Endothelial Cells

**Figure 4:**
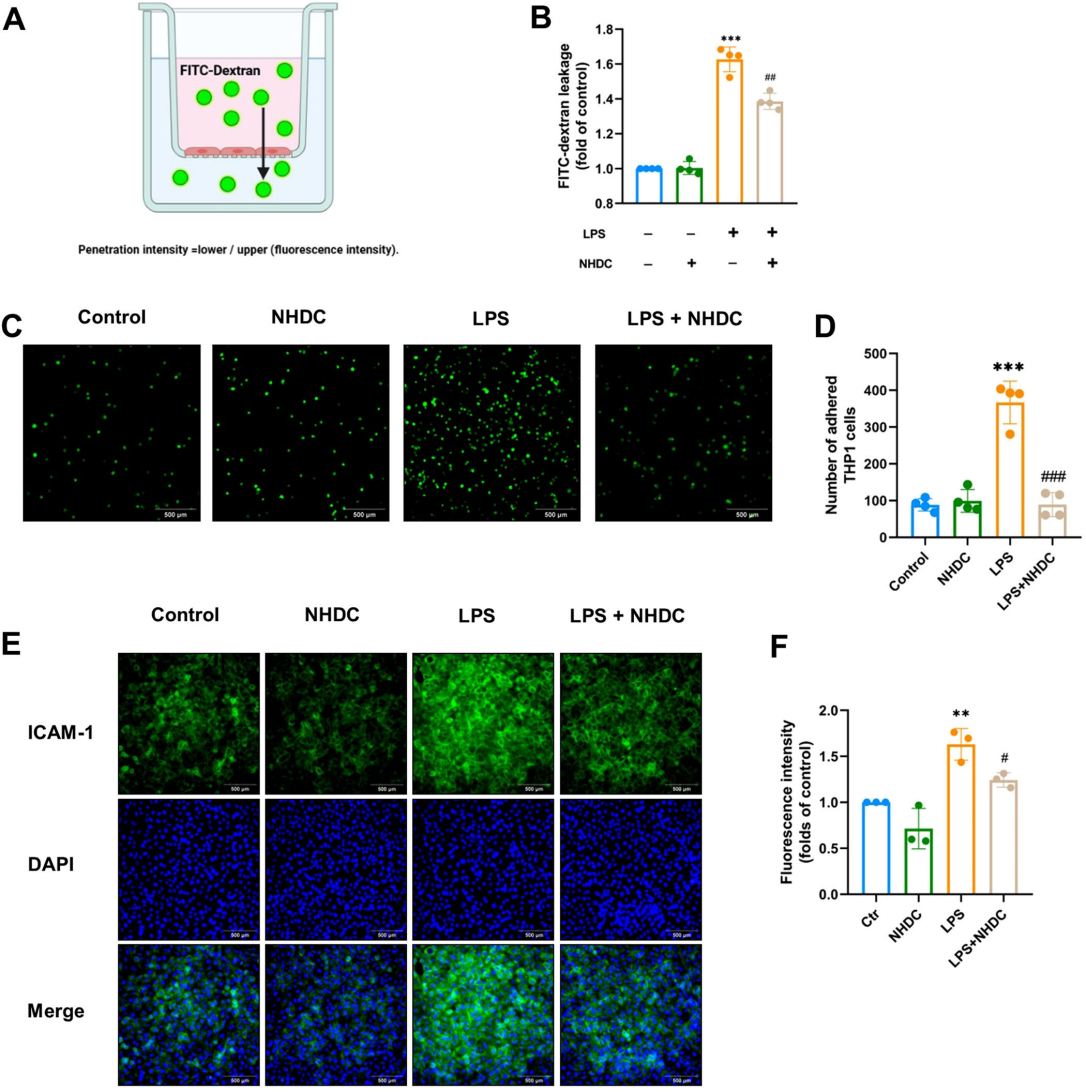
Effects of NHDC on permeability and adhesion of endothelial cells in vitro. (A, B) Schematic diagram and statistical analysis of HUVEC penetration test in vitro (*n* = 4). (C) Fluorescence microscopy of THP1 cells (labeled with 5 uM Calcein AM) incubated with HUVEC cells for 2 h. (D) Quantification of THP1 adhesion to HUVEC cells (*n* = 4). (E) Immunofluorescence staining of ICAM‐1 in different groups. (F) Immunofluorescence intensity analysis of ICAM‐1 (*n* = 3). Scale bar: 500 μm; **p < 0.01, ***p < 0.001, ^#^ *p* < 0.05, ^##^ *p* < 0.01, ^###^ *p* < 0.001; *Compared with control group, #compared with LPS group.

To explore the effect of NHDC on the functional integrity of endothelial cells exposed to LPS, we tested the permeability of HUVEC after LPS treatment using FITC‐dextran permeation in vitro experiments as shown in Figure 4A. LPS treatment for 24 h significantly increased the permeability of HUVEC compared to the control, while the NHDC group pretreated for 2 h significantly reduced it (*p* = 0.001) (Figure 4B). LPS‐induced vascular endothelial barrier dysfunction is often accompanied by an increase in endothelial inflammatory adhesion [19]. This phenomenon is determined by the defensive and structural properties of endothelial cells. In an inflammatory state, these cells recruit immune cells from the bloodstream to infiltrate the endothelial barrier and reach inflamed tissue sites [20, 21]. Thus, we further investigated the influence of NHDC on the adhesion of LPS‐stimulated HUVEC cells fluorescence microscopy by detecting their adhesion to THP‐1. Our results show that LPS significantly enhanced the adhesion ability of HUVEC, while NHDC effectively mitigated this effect (*p* < 0.001) (Figure 4C,D). Additionally, immunofluorescence experiments revealed that NHDC downregulated the expression of adhesion protein ICAM‐1 compared to LPS alone. These results suggest that pretreatment of NHDC may preserve endothelial barrier function by reducing endothelial cell adhesion during inflammatory states (Figure 4E,F).

### 3.5 NHDC Alleviates LPS‐Induced Oxidative Stress and Inflammatory Responses in Endothelial Cells In Vitro

**Figure 5:**
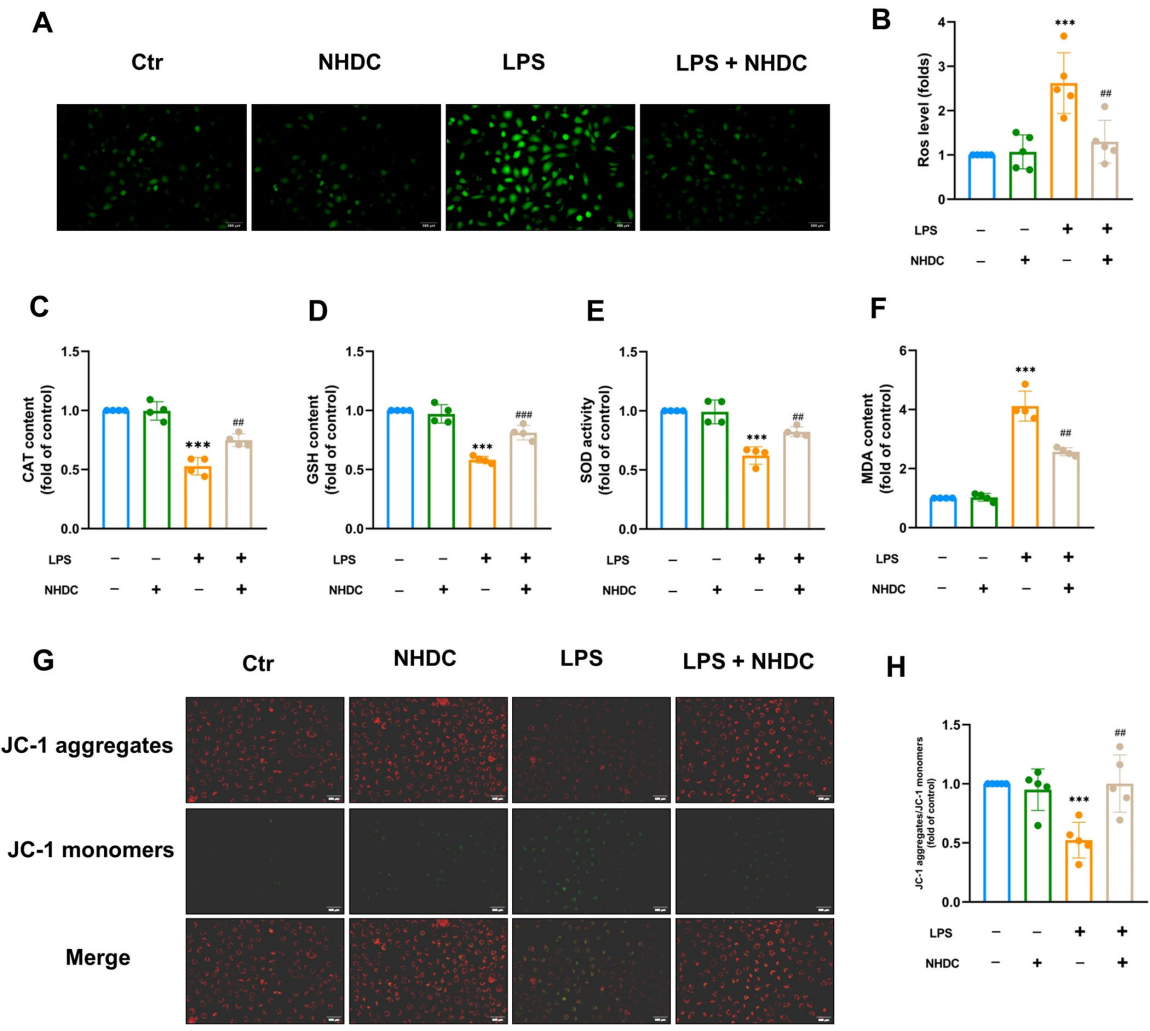
Effects of NHDC on total antioxidant capacity of HUVEC cells exposed to LPS. (A) Reactive oxygen species levels in HUVEC cells after different treatments assessed using DCFH‐DA. (B) Statistical analysis of ROS level (*n* = 5). (C–F) CAT, SOD, GSH, and MDA activities of HUVEC in different groups (*n* = 4). (G) Fluorescence imaging of MMP levels in different groups of cells after JC‐1 staining. (H) Statistical analysis of mitochondrial membrane potential levels (*n* = 5). Scale bar: 500 μm; **p < 0.01, ***p < 0.001, ^#^ *p* < 0.05, ^###^ *p* < 0.001; *Compared with control group, #compared with LPS group.

**Figure 6:**
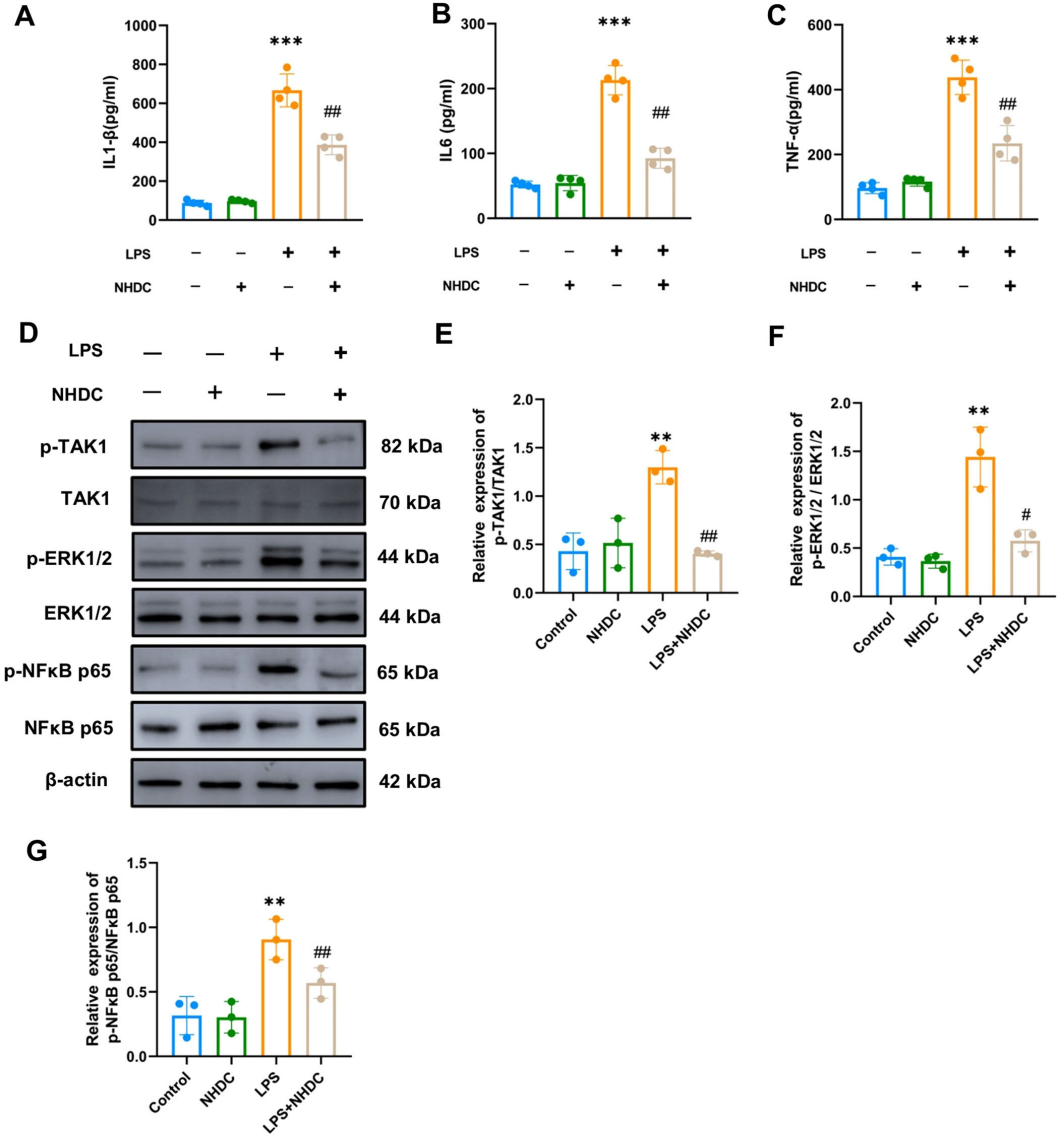
Effects of NHDC on inflammation regulation of HUVEC cells exposed to LPS. (A–C) The levels of IL‐1β, IL6, and TNF‐α released by HUVEC cells in the supernatant, assessed using ELISA (*n* = 4). (D) Western blot of TAK1, ERK1/2 phosphorylation levels, and NFκB expression. (E–G) Statistical analysis of western blot (*n* = 3). **p < 0.01, ***p < 0.001, ^#^ *p* < 0.05, ^##^ *p* < 0.01; *Compared with control group, #compared with LPS group.

To further explore the protective mechanism of NHDC, we investigated its effects on oxidative stress, which plays a key role in endothelial dysfunction during sepsis. During sepsis, endothelial cells produce excessive ROS in response to toxin stimulation, leading to ROS‐induced damage [22]. We detected ROS levels in endothelial cells using a DCFH‐DA probe. The results showed that NHDC effectively reduced ROS levels in the LPS‐treated group (Figure 5A,B). Furthermore, it increased the expression levels of antioxidant reductase CAT, SOD, and GSH. Additionally, NHDC also reduced the production of MDA, an oxidative stress marker (Figure 5C–F). It is well known that mitochondria are major sources of ROS due to their role in cellular energy production and electron transport [23]. LPS alters the mitochondrial function of endothelial cells by decreasing the MMP [24]. However, we found that NHDC treatment effectively counteract the decline of MMP caused by LPS (Figure 5G,H). Inflammatory imbalance is another consequence of oxidative imbalance in endothelial cells [25]. ELISA revealed that NHDC reduced the release of IL‐1β, IL‐6, and TNF‐α induced by LPS stimulation (Figure 6A–C).

### 3.6 NHDC Affects the Expression of TAK1, ERK1/2, and NF‐κB in Endothelial Cells In Vitro

Previous studies have shown that LPS can activate TAK1 and ERK1/2 in endothelial cells, promoting endothelial cell inflammation and increasing vascular permeability [26, 27]. Additionally, as a key inflammatory regulator, NF‐κB plays a pivotal role in regulating the onset and progression of sepsis [28]. To investigate this further, we detected the expression levels of these proteins through Western blot. The results showed that compared to control, LPS increased the phosphorylation levels of TAK1, ERK1/2, and NF‐κB. And NHDC reduces the expression of these inflammatory signaling pathways (Figure 6D–G).

### 3.7 Inhibition of NFKB on NHDC Protects Against LPS‐Induced Injury

**Figure 7:**
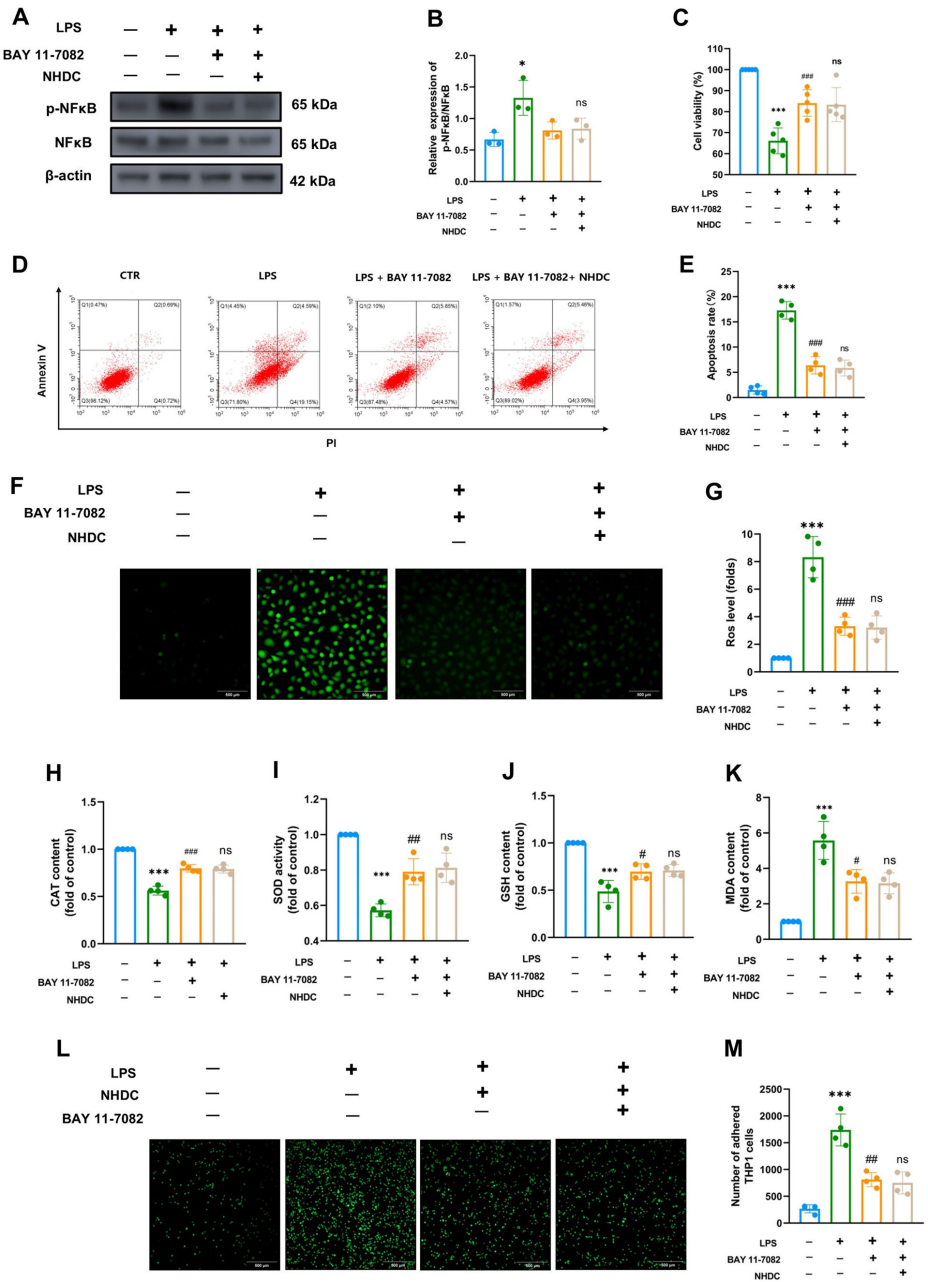
Effect of NFκB inhibition on LPS‐induced HUVEC damage. (A, B) Western blot and statistical analysis of NFκB phosphorylation levels after HUVEC‐treated with the inhibitor BAY11‐7082 (1 μM) for 24 h (*n* = 3). (C) CCK8 assay was used to detect HUVEC cell viability in different groups (*n* = 5). (D, E) flow cytometry and statistical analysis of apoptosis results in different groups of cells (*n* = 4). (F) Fluorescence representative map of ROS level in different groups of HUVEC detected by DCFH‐DA probe. (G) Statistical analysis of ROS levels (*n* = 4). (H–K) Activity levels of CAT, SOD, GSH, and MDA in different groups of HUVEC (*n* = 4). (L) Fluorescence representative plots of different groups of THP‐1 (labeled with 5 μM Calcein AM) adherent to HUVEC. (M) Quantitative statistics of THP‐1 adhesion (*n* = 4). Scale bar: 500 μm; *p < 0.05, ***p < 0.001, ^#^ *p* < 0.05, ^##^ *p* < 0.01, ^###^ *p* < 0.001; *Compared with control group, #compared with LPS group.

Furthermore, NFκB inhibitor BAY 11‐7082 was used to inhibit its activation (Figure 7A,B). Our results showed that BAY11‐7082 attenuated LPS‐induced inhibition of HUVEC viability, but there was no significant difference when BAY11‐7082 was combined with NHDC (Figure 7C). Flow cytometry results also showed that BAY11‐7082 slowed down LPS‐induced HUVEC apoptosis, and BAY11‐7082 combined with NHDC had no additional effect on HUVEC apoptosis (*p* > 0.05), as shown in Figure 7D,E. Further, in terms of antioxidant, BAY11‐7082 reduced the LPS‐induced generation of reactive oxygen species, enhanced the activities of antioxidant substances CAT, SOD, and GSH, and reduced the generation of membrane peroxidation product MDA (Figure 7F–K). BAY11‐7082 also inhibited LPS‐induced HUVEC adhesion to immune inflammatory cells, but no significant additional effect was observed when BAY11‐7082 was combined with NHDC (Figure 7L–M). To sum up, our results showed that NHDC attenuated LPS‐induced damage to HUVEC viability, oxidative capacity, and adhesion to inflammatory cells through NFκB‐dependent inhibition.

## 4 Discussion

Vascular permeability is an indicator of endothelial functional integrity. It is affected by various environmental factors, including mechanical damage and the breakdown of connecting structures [29]. Maintaining the integrity of endothelial cells is crucial for regulating vascular permeability [30]. Since microvessels are distributed throughout the body, endothelial damage can lead to systemic deterioration. The increased permeability of vascular endothelial cells not only intensifies the local tissue inflammation but also leads to various adverse events such as disturbances in water and electrolyte balance and decreased blood volume. As a result of direct contact with endotoxins, endothelial cell apoptosis is very common in sepsis. Especially in organs, damage to endothelial structures accelerates the further spread of infection, leading to organ damage [31]. In severe cases, these complications can culminate in shock [32, 33]. In our study, we found that pretreatment with NHDC can reduce the permeability of blood vessels and preserve vascular integrity through in vivo and in vitro experiments. One of the most important manifestations is that the pretreatment of NHDC can reduce apoptosis and avoid structural damage to endothelial cells.

Oxidative stress and an exacerbated inflammatory response are other important mechanisms of endothelial injury. Sepsis induces a paradoxical increase in free radical oxygen and nitrogen compounds following hypoxia, exceeding the capacity of nonenzymatic and enzymatic antioxidant systems and triggering oxidative stress [34, 35]. Mitochondria are the primary sites of ROS production and are particularly vulnerable to oxidative damage [36]. Excessive ROS production can damage polyunsaturated fatty acids, leading to lipid peroxidation. This compromises mitochondrial membrane integrity and impairs the activity of biological enzymes. This leads to energy depletion within the cell, further accelerating cell damage and eventual death [37].

The activation of endothelial cells can trigger an immune response, activating the endogenous immune system and leading to multiorgan failure [38]. Endothelial damage releases harmful substances, including ROS, inflammatory mediators, and immune‐activating factors. This triggers a series of pathophysiological reactions, such as cytokine storm, oxidative stress, inflammation, and coagulation dysfunction [3, 4]. Pretreatment of NHDC reduces the release of inflammatory mediators in endothelial cells. Further investigation suggests that this may occur through its effects on the phosphorylation levels of TAK1 and ERK1/2, as well as through inhibition of the activity of the NF‐κB signaling pathway, which plays a significant role in the regulation of inflammatory responses. Inhibition of NF‐κB significantly ameliorated LPS‐induced endothelial damage. However, NHDC treatment did not produce an additional effect, suggesting that NHDC exerts its protective effect primarily by inhibiting NF‐κB activation.

However, our study has several limitations. First, we used intraperitoneal injection of LPS in mice to model the internal environment of sepsis in vivo. While this is a commonly used method for sepsis modeling, it does not fully replicate the complex disease conditions observed in human sepsis [39]. Therefore, a small‐scale clinical trial should be considered in the next stage. Although NHDC has not yet reported data on the treatment of human diseases, as mentioned earlier, extensive data indicate that NHDC is highly safe [4, 5]. In addition to being a widely used artificial sweetener, NHDC is also used as an excipient in medications, such as sucrose hydroxyl ferric oxide, a phosphate‐reducing drug commonly prescribed for chronic kidney disease [40]. Furthermore, optimizing the drug delivery of NHDC is an important area that requires attention in future clinical applications. Since no reports currently exist regarding the intravenous use of NHDC, we utilized oral gavage in our study. Future research should focus on optimizing NHDC's properties, such as modifying functional groups to enhance water solubility, to improve its delivery to the vascular system [41].

## 5 Conclusions

Our study demonstrated that pretreatment with the artificial sweetener NHDC effectively alleviates LPS‐induced organ damage and vascular endothelial dysfunction. Further studies suggest that it may reduce the inflammatory response in endothelial cells by lowering permeability, enhancing their antioxidant capacity, and inhibiting the phosphorylation of the TAK1, ERK1/2, and NF‐κB signaling pathways.

## Author Contributions

**Yuxin Nong:** conceptualization, data curation, formal analysis, methodology, visualization, writing–original draft. **Junquan Lu:** data curation, formal analysis, resources. **Danqing Yu:** conceptualization, funding acquision, project administration, formal analysis, methodology, supervision, validation, writing–review and editing. **Xuebiao Wei:** conceptualization, data curation, formal analysis, methodology, resources, supervision, validation, writing–review and editing.

## Conflicts of Interest

The authors declare no conflicts of interest.

## Data Availability

The data sets are available from the corresponding author upon reasonable request.
